# Benign Pneumoperitoneum: A Case Report and Literature Review

**DOI:** 10.7759/cureus.115010

**Published:** 2026-08-23

**Authors:** Pooja Kumari, Nicola Brindley

**Affiliations:** 1 Paediatric Surgery, Temple Street Children's University Hospital, Dublin, IRL

**Keywords:** abdominal x-ray, bowel dystonia, neurodisability, pneumatosis coli, pneumoperitoneum

## Abstract

Pneumoperitoneum is almost always considered as a warning sign of hollow visceral perforation and requires an urgent surgical review. However, free air within the peritoneal cavity does not always indicate a surgical emergency. In some cases, pneumoperitoneum can occur without true visceral perforation. Recognising this key difference is crucial to avoid unnecessary surgical intervention.

A six-year-old girl with GNAO1-related neurodevelopmental disorder, epilepsy, movement disorder, on percutaneous endoscopic gastrostomy (PEG) tube feeds, and an implanted deep-brain stimulator presented with recurrent seizures, irritability, and reduced well-being. A computed tomography (CT) scan of the abdomen was suggestive of significant pneumoperitoneum, extensive colonic pneumatosis, and dilated bowel loops. Despite these radiological findings, the patient remained clinically stable, with normal vital signs, a soft and non-tender abdomen, no signs of peritonitis, and unremarkable inflammatory markers.

The radiological imaging performed in this case contrasted with her clinical examination findings, which were quite reassuring for a benign pathology. Reports in the literature also describe similar cases with clinical presentations, where free intraperitoneal air arose from ruptured gas-filled cysts in the bowel wall rather than from actual perforation. Therefore, in patients who remain clinically stable, a cautious clinical approach with close monitoring and regular periodic reassessment is often more appropriate than immediate surgical intervention.

## Introduction

Pneumoperitoneum is usually considered a warning sign of hollow visceral perforation and often triggers an urgent surgical review. In everyday clinical practice, free air within the peritoneal cavity is commonly associated with serious intra-abdominal conditions, such as perforated viscus, bowel ischemia, necrotising enterocolitis (NEC), and sepsis. It is also a well-recognised feature in babies with gastroschisis. Because of this, it is often treated as a surgical emergency until proven otherwise. However, over recent years, it has become clear that not every case of pneumoperitoneum represents a visceral perforation, and some patients can be managed safely without surgery when the clinical picture is reassuring.

Benign pneumoperitoneum refers to the presence of intraperitoneal free air without a perforated viscus requiring surgical intervention. Several causes have been identified, including recent intra-abdominal procedures, use of mechanical ventilation, peritoneal dialysis, occurrence of thoracic barotrauma, percutaneous endoscopic gastrostomy (PEG)-related air leaks, and pneumatosis intestinalis [1-3]. 

Pneumatosis intestinalis is described as gas located within the bowel wall. It may affect any part of the bowel, the small intestine, the colon, or both. When the colon is predominantly involved, it is referred to as pneumatosis coli or pneumatosis cystoides coli [2], as demonstrated in our case. Although uncommon, this diagnosis is crucial because rupture of gas-filled cysts can release free air into the peritoneal cavity, creating pneumoperitoneum without true visceral perforation [2,4].

Imaging findings alone should not determine the clinical management. Findings such as abdominal tenderness, guarding, haemodynamic instability, fever, elevated inflammatory markers, and metabolic acidosis, or rising lactate, are more suggestive of significant underlying pathology and may warrant urgent surgical intervention [1,3,4]. In contrast, patients who appear clinically well, with a soft, non-tender abdomen and stable observations, can be managed with careful clinical as well as radiological monitoring and a conservative approach.

This distinction is especially important in children and in those with complex medical conditions. These patients may present atypically, as communication can be limited and challenging, and the surgery may carry a higher risk. Our case illustrates the difficulty of decision-making in a medically complex child who had marked pneumoperitoneum and colonic pneumatosis on imaging, yet showed very little clinical evidence to suggest an acute surgical abdomen.

## Case presentation

A six-year-old girl with a complex medical background was transferred from the District General Hospital (DGH) via Intensive Paediatric Team Assessment Services (IPATS) for further assessment. She is known to have a GNAO1-related neurodevelopmental disorder, associated with epilepsy, hyperkinetic movement disorder, global developmental delay, on PEG tube feeds, and a bilateral globus pallidus internus (GPI) deep brain stimulator inserted 12 months previously.

**Figure 1:**
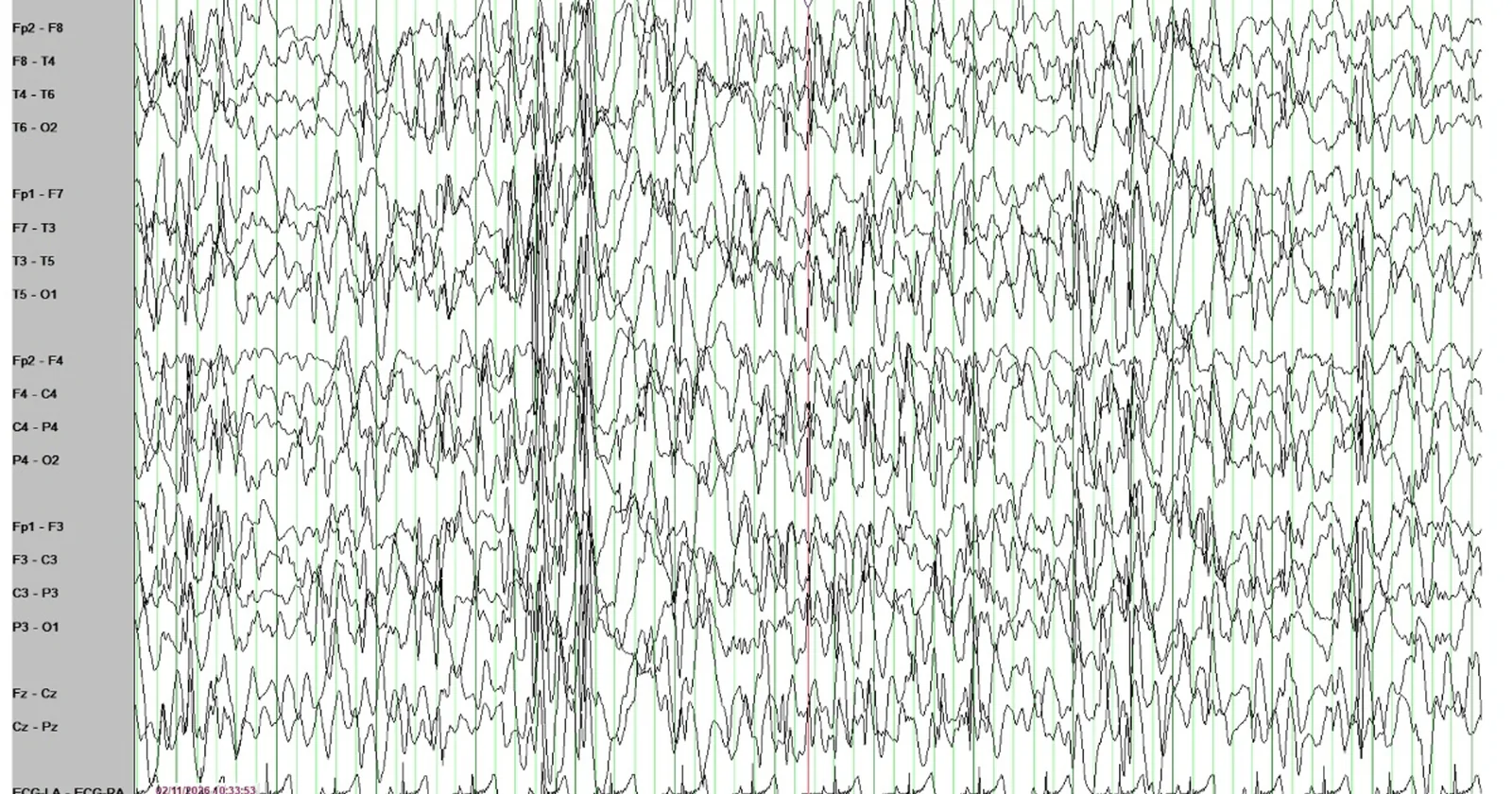
Interictal EEG showing a slow, chaotic and disorganised background with high-amplitude multifocal epileptiform discharges, giving a pattern of hypsarrhythmia Longitudinal bipolar montage. Sensitivity 10 uV/mm. Time base: 30 mm/sec.

She had been seizure-free for almost 5 years, and had been off anti-epileptic medication until 20 days before the current presentation. However, there had been concern about possible seizure recurrence during the preceding few months. An electroencephalogram (EEG) showed epileptic spasms (Figure 1), so she was restarted on antiepileptic drugs, i.e., levetiracetam, which was later changed to vigabatrin before being discharged home.

She was readmitted with further seizures and a general decline in her condition. Her mother described her as being “off form.” Changes were made to her anti-epileptic treatment, but she became very sleepy and flat. Nine days before the current presentation, she developed a 24-hour episode of vomiting. This was thought to be due to norovirus, common in the hospital at that time; there was no associated diarrhoea or fever, so she was discharged home again.

**Figure 2:**
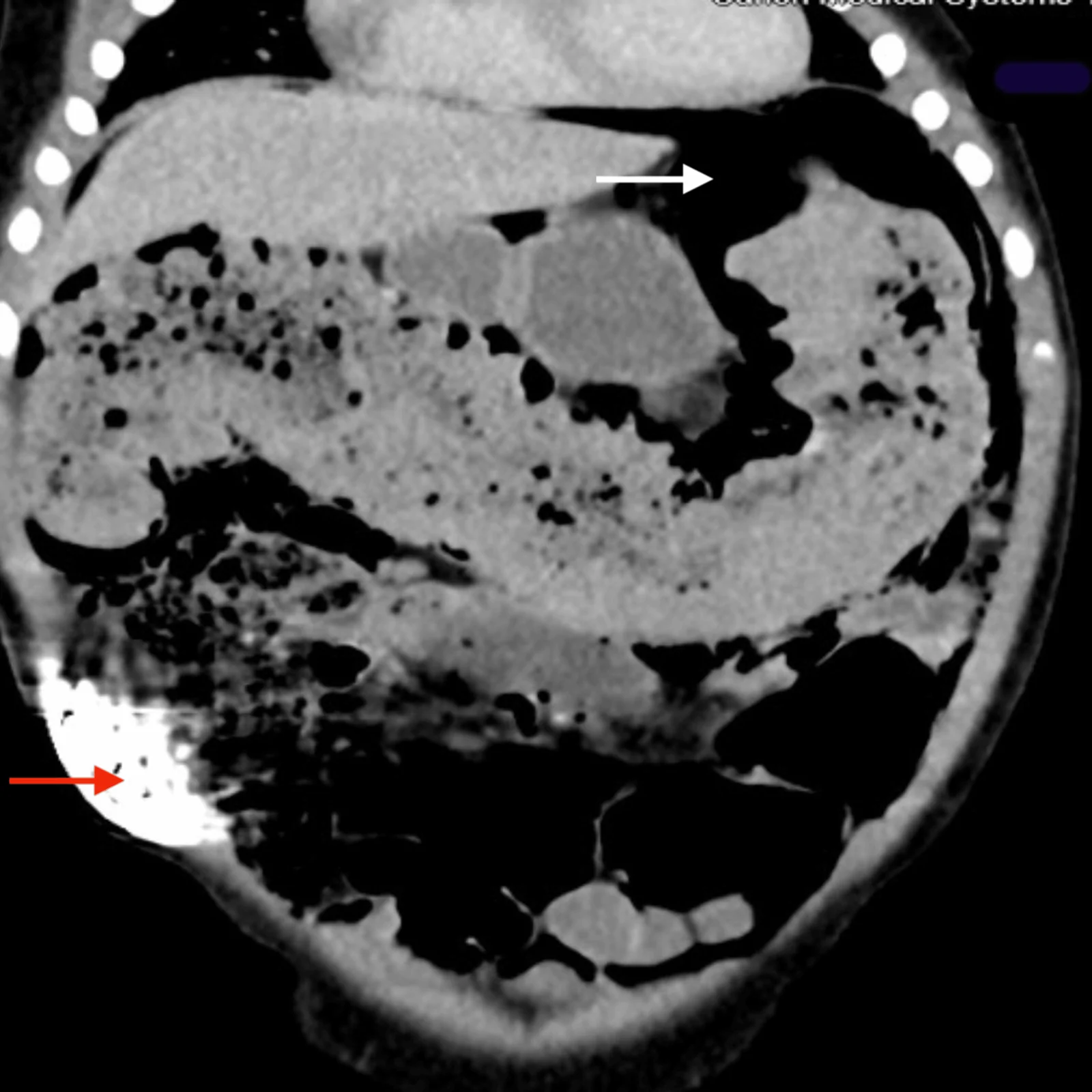
CT scan abdomen Coronal section showing large pneumoperitoneum (white arrow), diffusely dilated loops of the small and large bowel filled with faeces, pneumatosis coli, deep brain stimulator in place (red arrow), and no free fluid. The remainder of the abdominal and pelvic organs and osseous structures appear normal.

**Figure 3:**
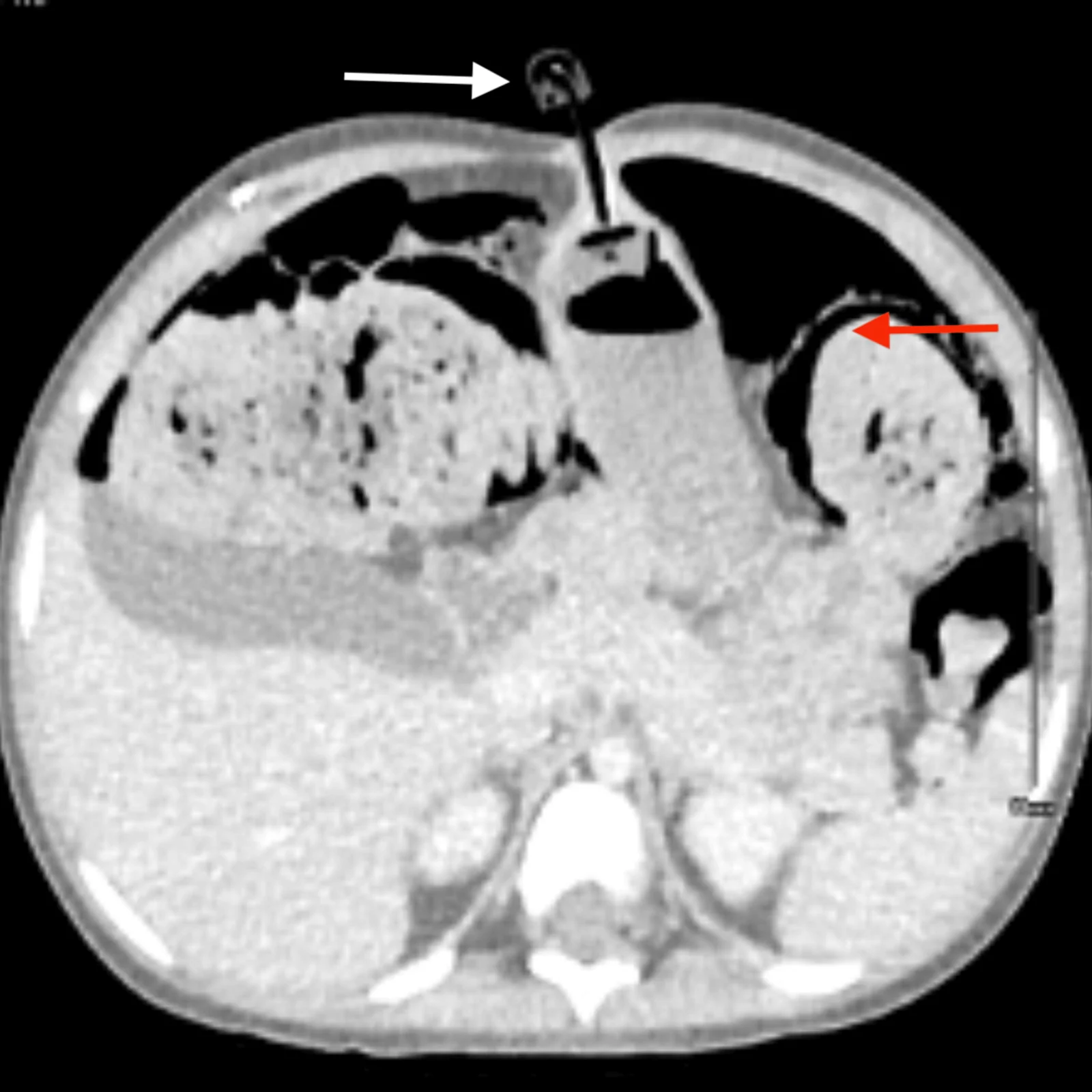
CT scan abdomen Axial image showing a large pneumoperitoneum, diffusely dilated loops of the small and large bowel filled with faeces, pneumatosis coli (red arrow), PEG tube in the stomach (white arrow) and no free fluid. PEG: percutaneous endoscopic gastrostomy

She re-presented with seizures, irritability, restlessness and ongoing parental concern. CT scan abdomen was performed because the child was non-verbal and her unexplained irritability raised the concern for occult intra-abdominal pathology. The CT scan of the abdomen and pelvis showed a large pneumoperitoneum, diffuse dilatation of both small and large bowel loops, a PEG tube in the stomach, and no free fluid (Figures 2-3). The scan also indicated extensive pneumatosis of the large bowel, a significant stool burden, and only a small amount of free fluid. Given the CT scan findings in a DGH, she was transferred to our hospital.

On arrival, she was haemodynamically stable. Oxygen saturations were 97% on room air, pulse 96-110 bpm, blood pressure 95/60 mmHg, respiratory rate 24 breaths/min, temperature 36.4 °C, and capillary refill time <2 seconds. 

She appeared pink and well-perfused. She was alert but agitated, with prominent athetoid movements. Her abdomen was full but soft, non-tender, with no guarding or signs of peritonism. The PEG site was clean and healthy. Gastric aspirate was minimal, clear, with small brown flecks and no bile. She was passing flatus during the examination. Per rectal examination revealed an empty rectum. Inflammatory markers remained normal. Her C-reactive protein (CRP) was <4 (0-10 mg/dl) and white cell count (WBC) was 10.9 (4.5 to 13.5 x 10^9^/L). Blood gas parameters, serum lactate levels, electrolytes, and renal function were all within normal limits.

Given her stability and lack of peritonism, the plan was made to manage her conservatively. She was kept nil per os (NPO), the PEG tube was kept on open drainage, intravenous (IV) fluids and antibiotics were started, and she was followed clinically and radiologically.

**Figure 4:**
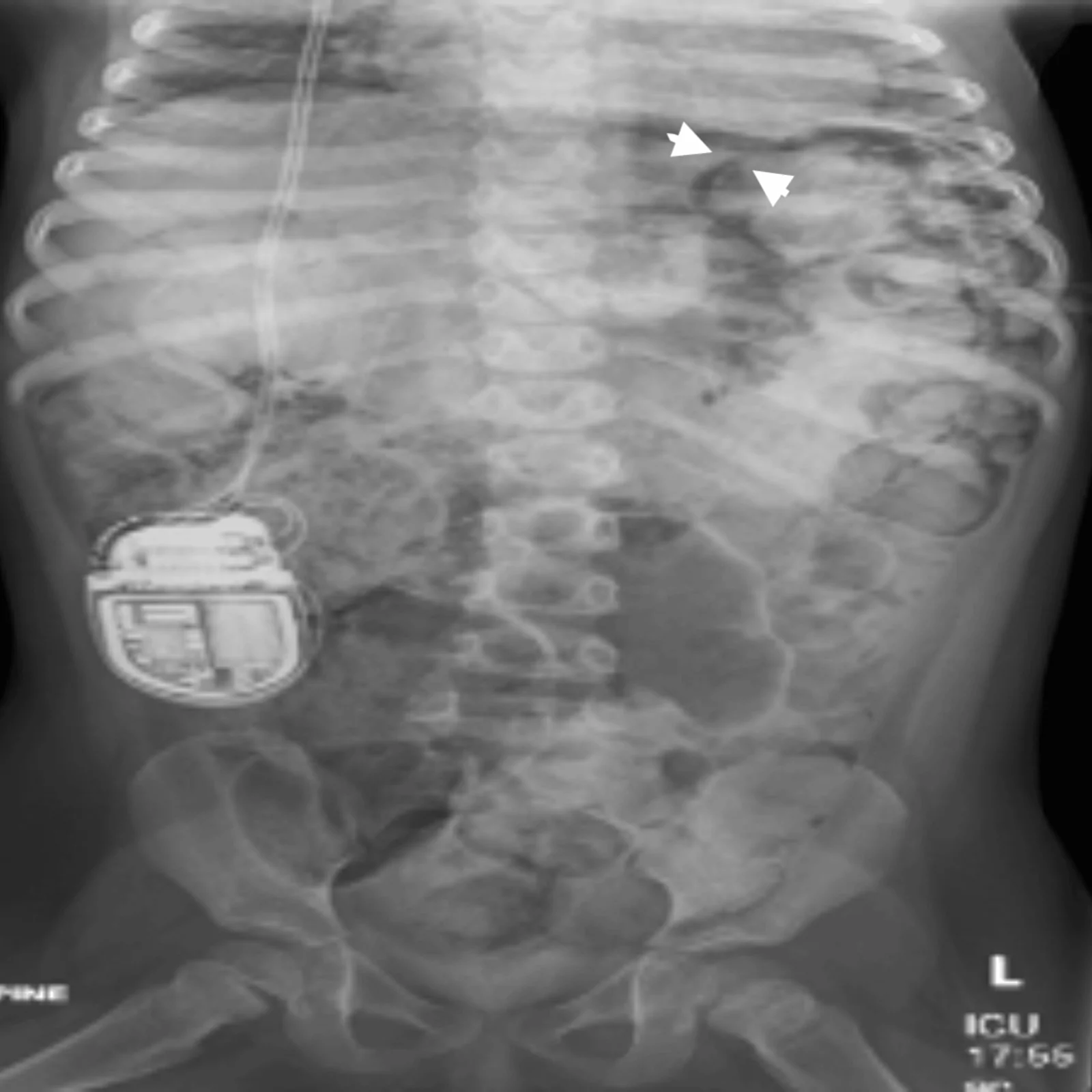
X-ray abdomen Day 2 post admission Positive Rigler sign (arrowheads) with evidence of pneumoperitoneum from extraluminal gas demonstrating a double-lumen pattern. Considerable amount of stool in the right and transverse colon. A deep-brain stimulator is demonstrated in the soft tissues of the right iliac fossa.

**Figure 5:**
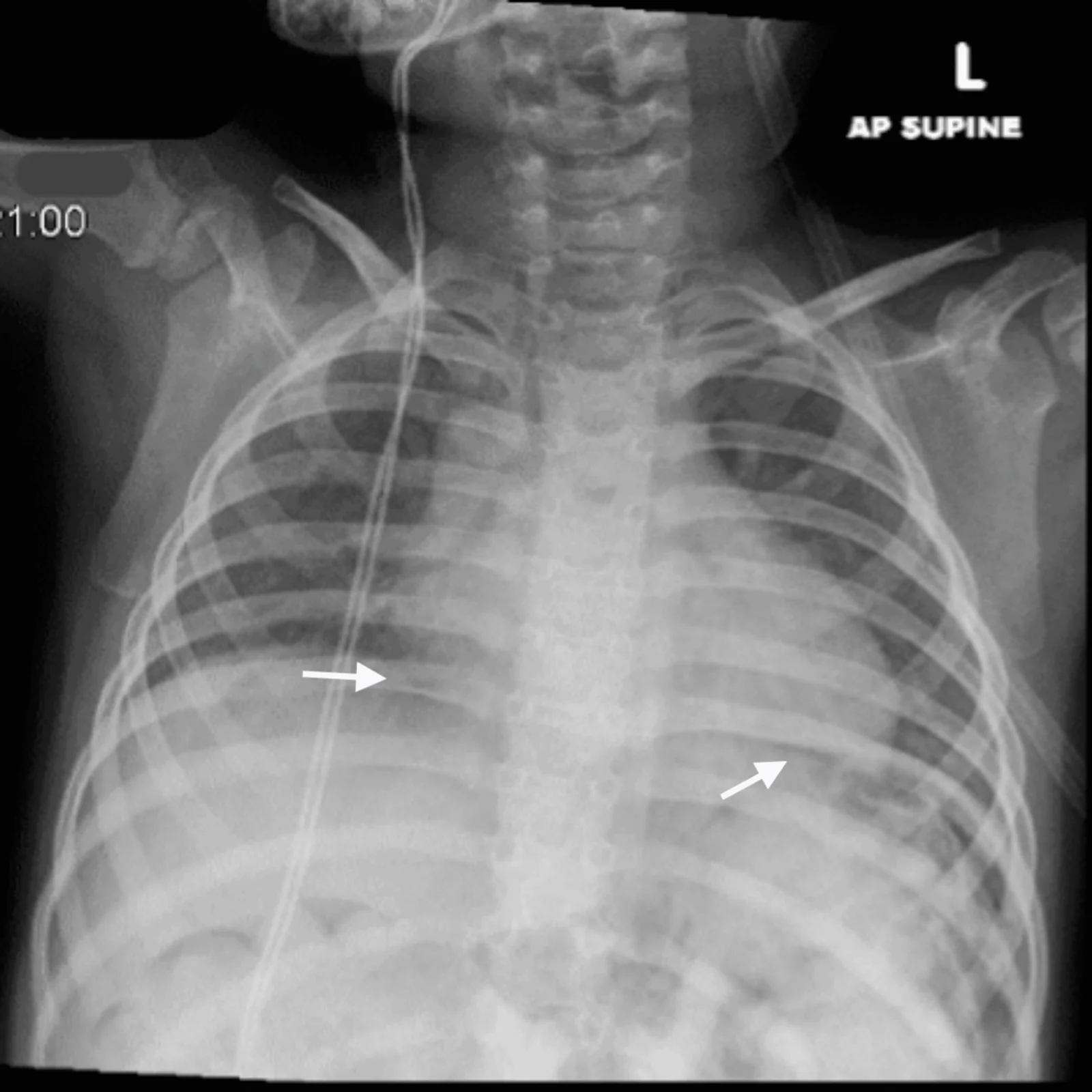
X-ray chest Day 2 post admission: free gas under both hemidiaphragms (white arrows), low lung volumes, no pleural effusion or pneumothorax

Plain abdominal and chest radiographs, performed the next day as a baseline, confirmed pneumoperitoneum with Rigler sign, pneumatosis of the large bowel, significant faecal loading in the right and transverse colon, free gas under both hemidiaphragms, low lung volumes, but no pleural effusion or pneumothorax (Figures 4-5).

**Figure 6:**
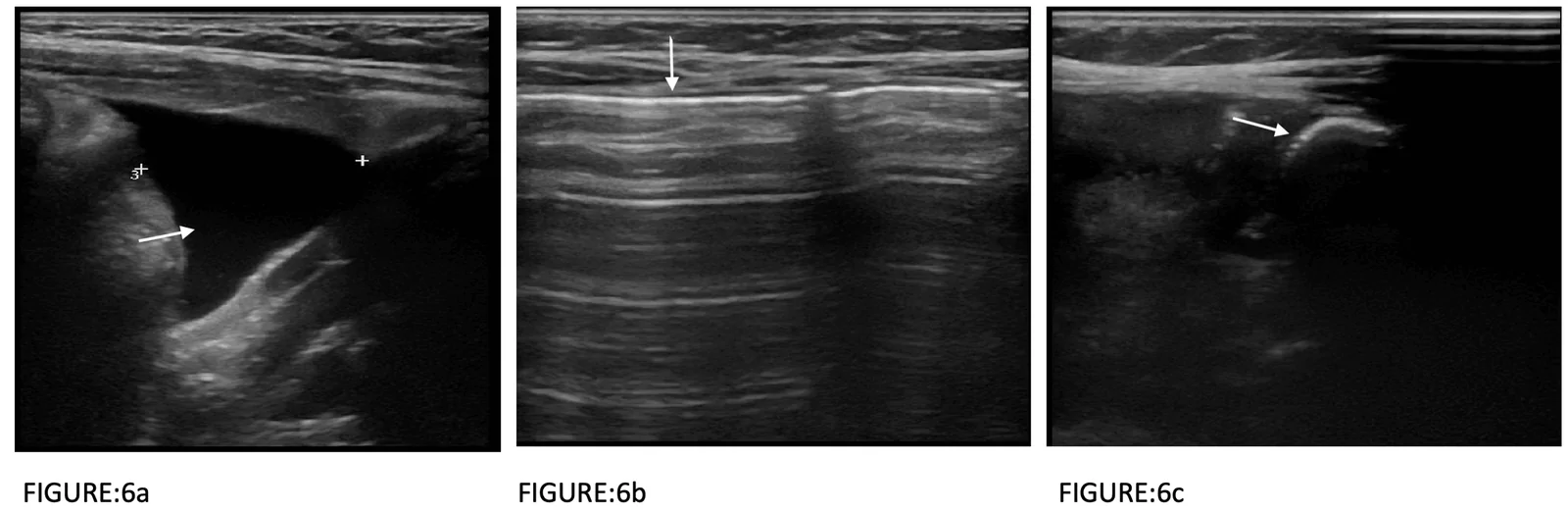
(a) A small amount of free fluid in the right lower abdomen (white arrow), (b) Pneumoperitoneum (white arrow), (c) Pneumatosis (white arrow), better evaluated on CT scan abdomen

**Figure 7:**
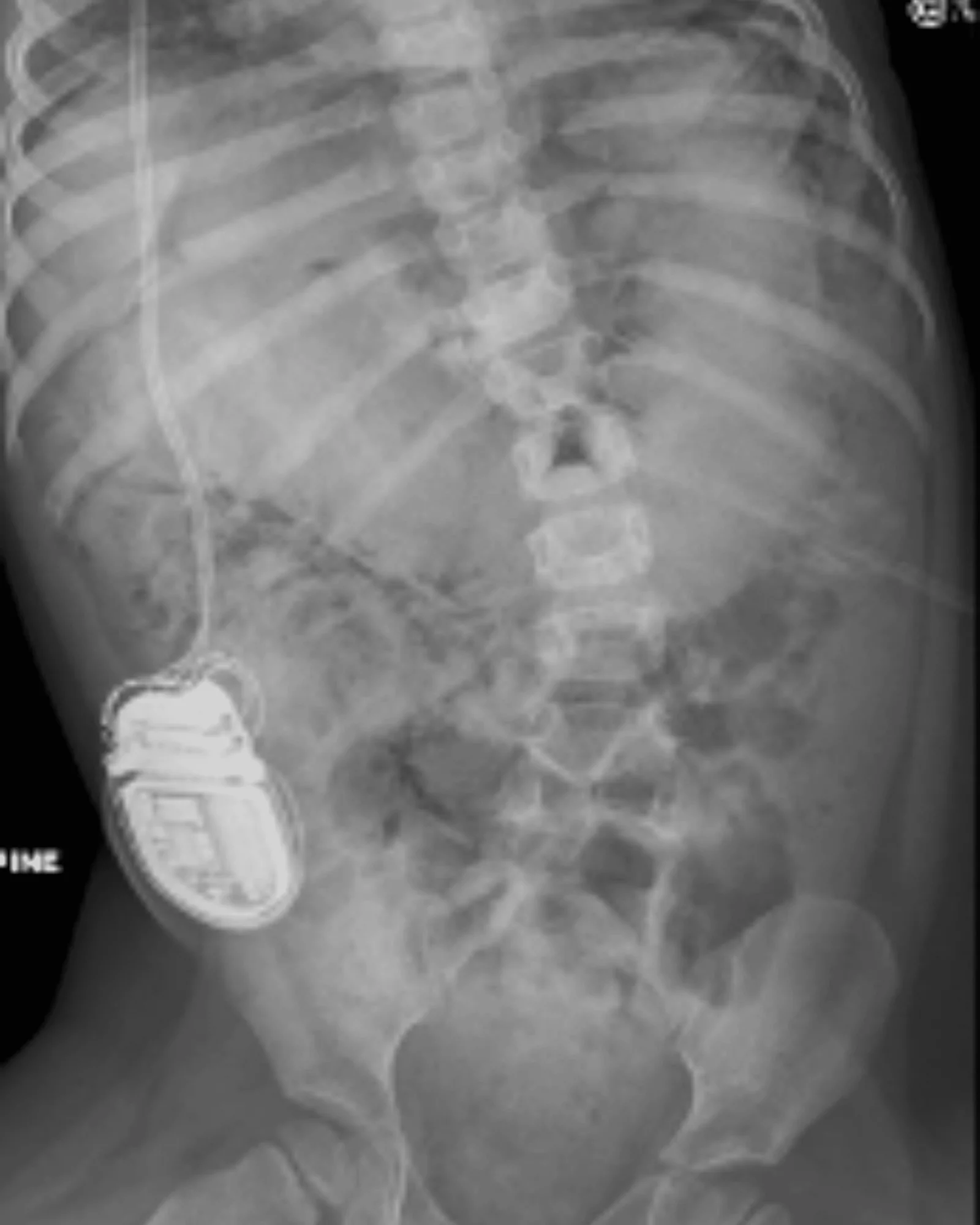
X-ray abdomen Day 3 post admission: persistent pneumoperitoneum projected over the liver, persistent impression of colonic pneumatosis, small bowel loops in the mid abdomen appear compressed, considerable amount of stool in the right transverse colon: overall improved appearance

**Figure 8:**
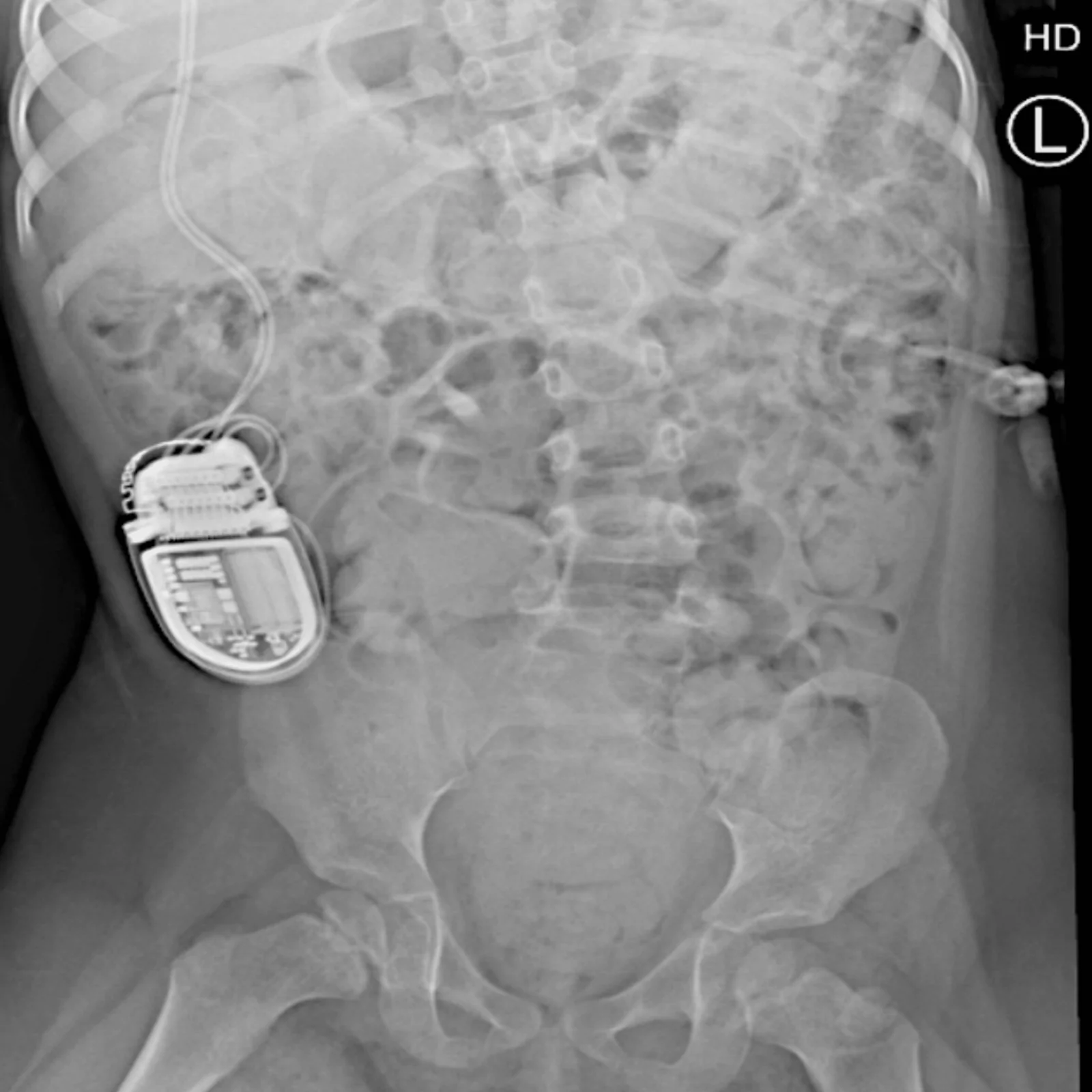
X-ray abdomen Day 4 post admission: small amount of persistent pneumatosis in the right flank; gastrostomy and deep brain stimulator noted

**Figure 9:**
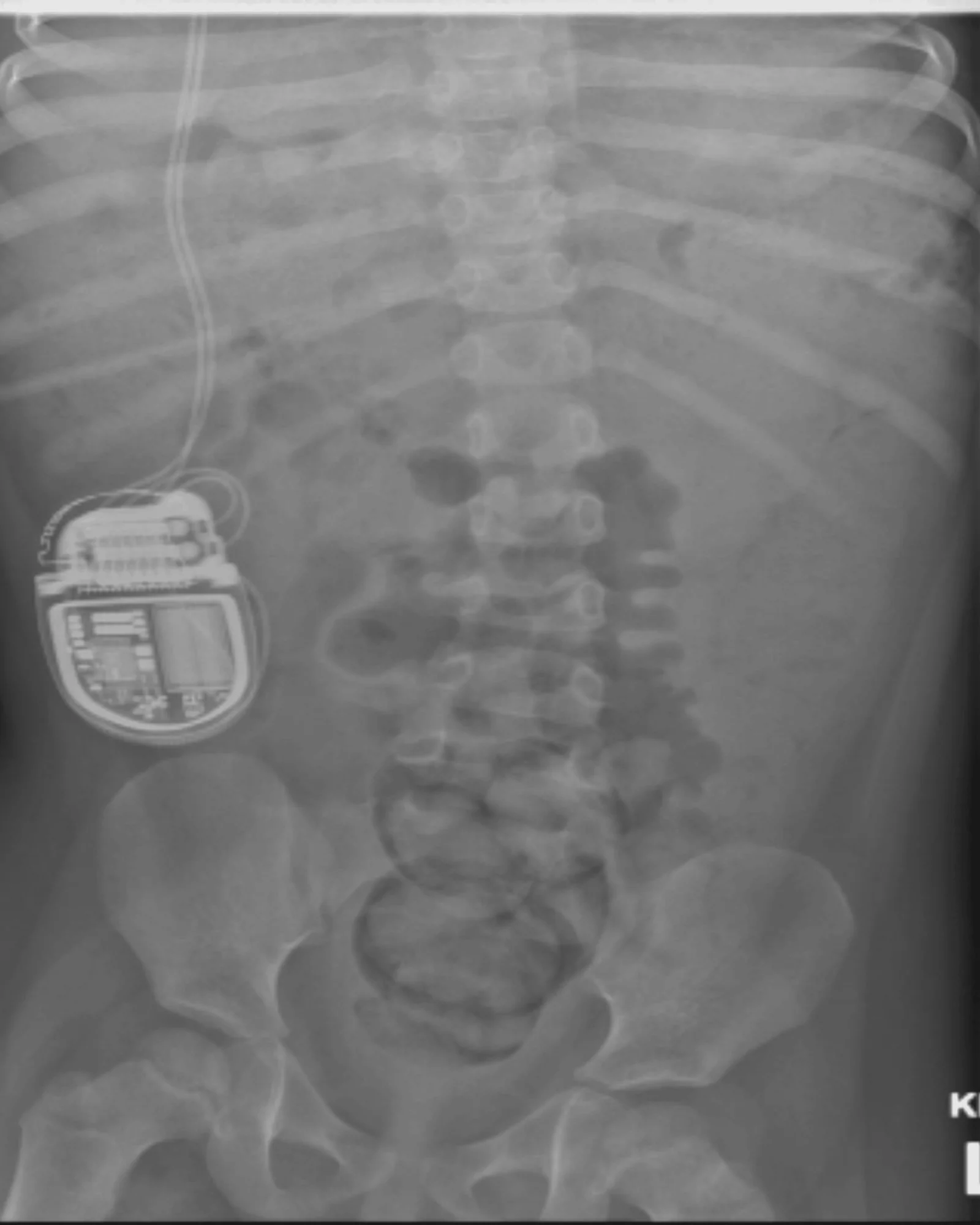
X-ray abdomen Day 5 post admission: no pneumatosis or pneumoperitoneum identified, no dilated loops; stool present in the descending colon

An ultrasound performed showed persistent pneumoperitoneum, transverse colon pneumatosis, decompressed small bowel loops, and ongoing stool burden (Figure 6). X-rays performed on subsequent days are shown in Figures 7-9.

**Figure 10:**
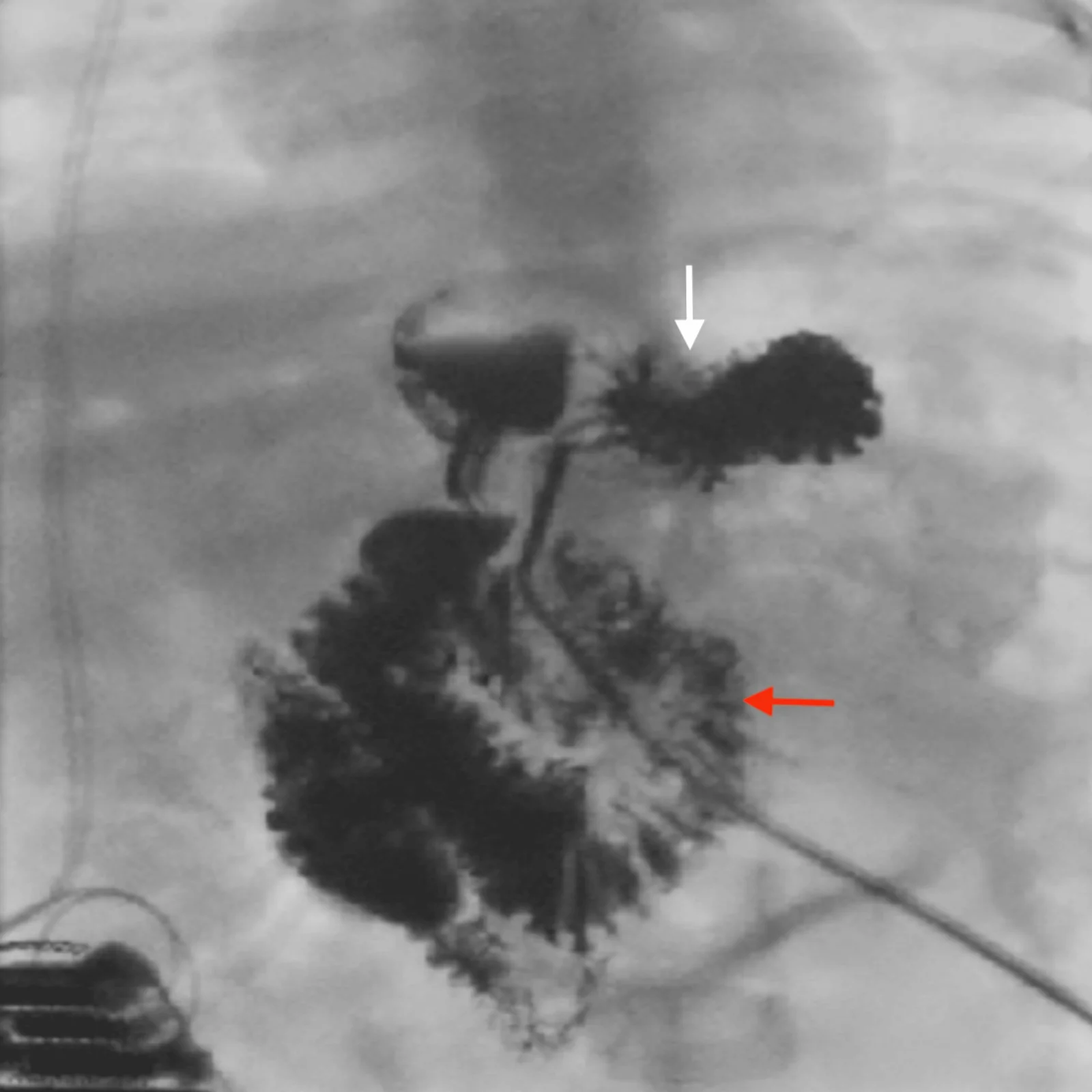
Contrast filling in the stomach (white arrow), suggesting the PEG tube position in the body of the stomach Contrast passes freely into the duodenum. The DJ stent lies to the left of the midline, and the jejunal loops (red arrow) lie centrally in the abdomen. PEG: percutaneous endoscopic gastrostomy

A tubogram performed the day after showed the gastric tube in place (Figure 10); feeds were restarted.

The child was observed until she was on full feeds, then discharged after medical review and with medical follow-up planned.

In summary, this was a medically complex child with dystonia, who presented with recurrent seizures and behavioural changes, marked pneumoperitoneum, and colonic pneumatosis on imaging, despite a soft, non-tender abdomen, no signs of peritonitis and normal inflammatory markers. This case represents a challenging balance between concerning radiological findings and a relatively reassuring clinical examination.

## Discussion

This case underlines the importance of assessing a patient based on the clinical presentation and physical examination rather than only relying on imaging. The CT scan revealed extensive pneumoperitoneum, Rigler sign (double wall sign), and significant colonic pneumatosis (air within the colonic wall). However, there were no signs suggestive of sepsis or peritonitis. The patient had a soft, non-tender abdomen and did not have elevated inflammatory markers and continued to pass flatus. This suggested that the abnormal CT scan findings were more consistent with a benign pneumoperitoneum caused by colonic pneumatosis rather than a perforated viscus.

Over many years, free air under the diaphragm had been considered as a surgical emergency. However, this may not be the case for all patients. The current literature indicates that the presence of pneumoperitoneum does not always indicate that there is a hollow visceral perforation [1,3]. Atri et al. describe a similar patient who had free air due to pneumatosis intestinalis but was treated conservatively because the patient remained clinically stable [1]. Sharp and Chuang pointed out that there is a substantial clinical range of pneumatosis intestinalis, ranging from asymptomatic benign cases to patients with bowel ischemia, and assessment via clinical examination is the most important differentiating factor [3].

Pneumatosis intestinalis is a challenging cause, as its radiological appearance can be dramatic and it can be easily mistaken for surgical pathology. The exact mechanism for this pathology is unknown. But there are a few explanations, including raised intraluminal pressure and microscopic mucosal injury that allow luminal gas to track into the bowel wall; other explanations are the presence of gas-forming bacteria, and, in some cases, gas dissection from the thorax through tissue planes into the mesentery [2,4]. In many cases, multiple factors are usually involved. Bowel dysmotility in patients with dystonia has also been recognised as a contributing factor [5,6].

Our patient had reassuring clinical features, i.e., no guarding or rebound tenderness, no signs of haemodynamic instability, and all the inflammatory markers were within normal limits, making significant bowel ischemia or contamination of the peritoneal cavity very unlikely. Furthermore, the bowel function appeared to be intact; the patient was passing flatus and did not appear to be experiencing evidence of bowel obstruction.

Constipation may have been a contributing factor to the underlying cause of the pneumatosis coli [2]. Imaging demonstrated a significant amount of faecal material present in both the right colon and transverse colon, which may account for the high degree of increased intraluminal pressure (due to constipation and distended bowel). This is a recognised mechanism by which pneumatosis coli can develop [2]. Pneumatosis coli may develop when gas dissects through small defects in the mucosa and collects within the wall of the bowel. Due to the rupture of subserosal cysts, pneumoperitonitis may result in abscess formation without true perforation of the bowel [2,4]. The presumed explanation in our case is pneumoperitoneum secondary to pneumatosis coli without evidence of full-thickness bowel perforation; however, the precise mechanism cannot be definitively established in the absence of surgical or pathological confirmation.

It is also possible that her underlying neurodevelopmental condition may have played a role. Many children with neurodevelopmental disabilities frequently have chronic constipation, decreased gut motility, limited mobility, and bowel dystonia, and are dependent on enteral nutrition, which puts them at risk for developing distended bowels and abnormal gas handling [5,6]. In addition, due to the unique characteristics of many patients seen in this population, the ability to interpret symptoms presents greater difficulty in obtaining a clinical assessment for a specific condition.

The aforementioned case illustrates the significance of a multidisciplinary team approach to management, as well as the utility of serial assessments. If the radiographic findings of a patient are suggestive of some form of pathology, but the patient has a stable clinical status, the presence of ongoing observation, serial abdominal examination, and laboratory study results trending over time may be more significant than an immediate surgical intervention. Surgical management should only be used for patients who experience significant clinical deterioration, peritonitis, metabolic acidosis, an increase in inflammatory markers, or other signs consistent with possible bowel perforation or bowel ischemia.

Overall, this case adds to the growing recognition that benign pneumoperitoneum is a genuine clinical entity. Awareness of this condition is important for reducing unnecessary laparotomies, particularly in medically complex paediatric patients, while still ensuring timely intervention for those with true surgical pathology.

## Conclusions

This case illustrates that extensive pneumoperitoneum visualised on imaging may not always suggest that there is an acute surgical abdominal pathology. Despite the impressive radiology findings, the patient continued to be well, had no evidence of peritonitis, and had normal inflammatory markers, suggesting that this was a benign process due to colonic pneumatosis.

The importance of correlating imaging findings with clinical evaluation and repeated physical examination, particularly in children with complicated medical problems and neurodisability, is emphasised by this case. A balanced, patient-centred approach will assist in minimising the number of avoidable procedures, while also allowing for the appropriate recognition of those patients who have true surgical pathology.
